# Resource allocation during the coronavirus disease 2019 pandemic and the impact on patients with lung cancer: a systematic review

**DOI:** 10.1093/icvts/ivad190

**Published:** 2023-11-28

**Authors:** Ryaan EL-Andari, Nicholas M Fialka, Uzair Jogiat, Bryce Laing, Eric L R Bédard, Jayan Nagendran

**Affiliations:** Division of Cardiac Surgery, Department of Surgery, University of Alberta, Edmonton, AB, Canada; Division of Cardiac Surgery, Department of Surgery, University of Alberta, Edmonton, AB, Canada; Division of Thoracic Surgery, Department of Surgery, University of Alberta, Edmonton, AB, Canada; Division of Thoracic Surgery, Department of Surgery, University of Alberta, Edmonton, AB, Canada; Division of Thoracic Surgery, Department of Surgery, University of Alberta, Edmonton, AB, Canada; Division of Cardiac Surgery, Department of Surgery, University of Alberta, Edmonton, AB, Canada

**Keywords:** Coronavirus disease 2019, Lung cancer, Resource allocation, Pandemic

## Abstract

**OBJECTIVES:**

The coronavirus disease 2019 (COVID-19) pandemic resulted in unprecedented tolls on both economies and human life. Healthcare resources needed to be reallocated away from the care of patients and towards supporting the pandemic response. In this systematic review, we explore the impact of resource allocation during the COVID-19 pandemic on the screening, diagnosis, management and outcomes of patients with lung cancer during the pandemic.

**METHODS:**

PubMed and Embase were systematically searched for articles investigating the impact of the COVID-19 pandemic on patients with lung cancer. Of the 1605 manuscripts originally screened, 47 studies met the inclusion criteria.

**RESULTS:**

Patients with lung cancer during the pandemic experienced reduced rates of screening, diagnostic testing and interventions but did not experience worse outcomes. Population-based modelling studies predict significant increases in mortality for patients with lung cancer in the years to come.

**CONCLUSIONS:**

Reduced access to resources during the pandemic resulted in reduced rates of screening, diagnosis and treatment for patients with lung cancer. While significant differences in outcomes were not identified in the short term, ultimately the effects of the pandemic and reductions in cancer screening will likely be better delineated in the coming years. Future consideration of the long-term implications of resource allocation away from patients with lung cancer with an attempt to provide equitable access to healthcare and limited interruptions of patient care may help to provide the best care for all patients during times of limited resources.

**Figure:**
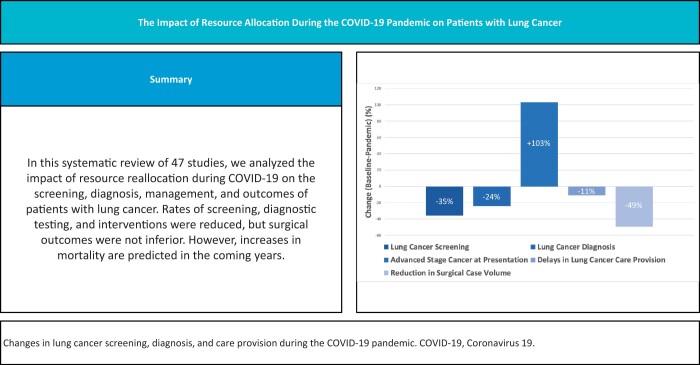

## OBJECTIVES

Lung cancer is among the leading causes of death worldwide and is the leading cause of cancer deaths [1]. With an incidence of 11.4% of new cancer cases and annual cancer mortality of 18.0%, lung cancer carries a significant burden on patient survival, quality of life, and strain on the healthcare system [1]. Despite this, early detection of resectable disease is potentially curative. Surgery, in conjunction with chemotherapy, radiotherapy and in recent years, immunotherapy, form a cornerstone in the management of this complex disease [1–3].

The severe acute respiratory syndrome coronavirus 2 or coronavirus disease 2019 (COVID-19) pandemic was first encountered at the end of 2019. There have been 650 million infections, 6.6 million deaths and a surge in hospital admissions that overwhelmed healthcare systems globally since the first reported cases in 2019 [4]. In an attempt to respond to the increasingly limited resources available, governments and policymakers were required to make decisions regarding resource allocation. As transmission characteristics, mutagenicity and short- and long-term outcomes were largely unknown, there was understandable caution taken when making these decisions during the early phases of the COVID-19 pandemic [5–7]. Cautionary resource allocation resulted in personal protective equipment, ventilators, hospital beds and staffing being redirected towards the pandemic response.

Surgical programs internationally were significantly impacted by resource redirection and constraints [5, 6]. In many countries and across a broad range of specialties, elective cases were reduced or halted altogether at times, with only emergent cases proceeding and patients otherwise being placed on ever-growing waiting lists. The resulting delay in care for patients who needed non-emergent care caused disease progression, potentially inferior surgical outcomes, and has tragically resulted in the progression to unresectable disease in some cases [2, 8].

Considering the necessity of early detection and intervention, patients with lung cancer are at increased risk for adverse outcomes with delayed screening, diagnosis and treatment [5, 8, 9]. Upstaging of early to advanced-stage lung cancer has the potential to significantly reduce a patient’s survival and impair their quality of life [5]. The purpose of this systematic review was to consolidate and appraise the literature investigating resource allocation during the COVID-19 pandemic and to explore the impact of resource allocation on the diagnosis, management and outcomes of lung cancer.

## METHODS

### Literature search strategy

Two authors systematically searched the PubMed and Embase databases. The search terms used were ‘lung, thoracic, cancer, malignancy, oncology, COVID-19, pandemic, screening, surgery, delay’ individually or in combination. The search was conducted including literature published from 1 January 2019 to 30 November 2022. The reference lists of identified manuscripts were screened to identify additional articles. Data were extracted from articles by 2 authors based on prespecified outcomes as detailed below.

### Eligibility criteria and data extraction

**Figure 1:**
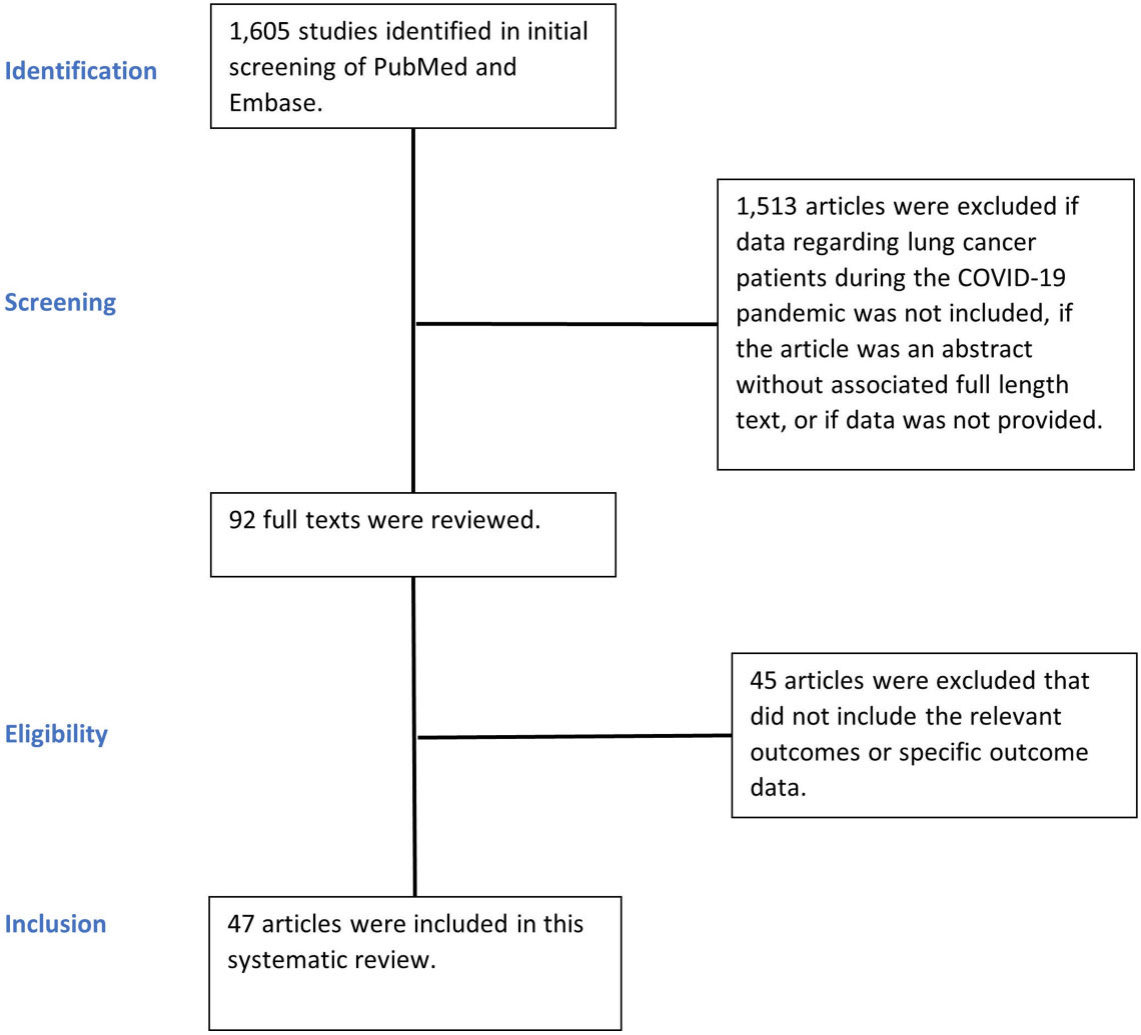
Study flow chart based on the Preferred Reporting Items for Systematic Reviews and Meta-Analyses guidelines.

The Preferred Reporting Items for Systematic Reviews and Meta-Analyses guidelines and a published explanation of the Preferred Reporting Items for Systematic Reviews and Meta-Analyses guidelines were referenced in structuring and conducting this systematic review [10, 11]. This review included retrospective and prospective studies examining the toll of resource allocation during the COVID-19 pandemic on the screening, diagnosis, management and outcomes of patients with lung cancer internationally. The inclusion criteria for this systematic review were studies published since 2019 investigating screening, diagnosis, management and postoperative outcomes of patients with lung cancer in relation to resource allocation and reduced available resources as a result of the COVID-19 pandemic. Case reports or series, reviews, abstracts without an associated full text, studies focusing on an era prior to the COVID-19 pandemic or studies investigating outcomes of conditions other than lung cancer were excluded from this study. Initial screening of 1605 manuscript titles and abstracts was performed by 2 authors. Once relevant manuscripts were identified, 92 full texts were reviewed, and 47 were included in this review (Fig. 1 and Table 1).

**Table 1:** Characteristics of included studies

Study name	Study type	Study organization	Years data collected	Study population	Location	Outcomes
Bertolaccini and Spaggiari [39]	Retrospective, single centre	Hub-and-spokes system for oncological care	2019–2020	Patients with thoracic malignancy	Italy	Thoracic oncologic surgical activity
Bertolaccini *et al.* [23]	Retrospective, single centre	Group 1: baseline (January–July 2019) Group 2: January–July 2020 Group 3: January–July 2021	2019–2021	Lung cancer patient undergoing lobectomy	Italy	Disease stage
Bhalla *et al.* [29]	Prospective, multicentre	Group 1: baseline (December 2019) Group 2: pandemic period (July 2020)	2019–2020	Adult patients with a current or history of hematological malignancy or invasive solid tumour	USA	Cancer care delivery (number of in-person outpatient visits, telehealth visits and pandemic-related delays of oncologic care)
Cantini *et al.* [5]	Retrospective, multicentre	Group 1: baseline (2019) Group 2: pandemic (2020)	2019–2020	Newly diagnosed lung cancer patient	Italy	Lung cancer diagnosis and access to treatment
Ciriaco *et al.* [38]	Retrospective, single centre	Description of COVID-related changes in NSCLC treatment	2019–2020	Patients with NSCLC lung cancer	Italy	Thoracic oncologic surgical activity
Conibear *et al.* [2]	Retrospective, multicentre	Group 1: baseline (2019)—33 091 patients Group 2: pandemic (2020)—31 371 patients	2019–2020	Patients with lung cancer; RCRD	UK	Lung cancer diagnosis, stage at presentation, treatment, survival
Degeling *et al.* [47]	Population-based modeling study	Baseline scenario Stage shift scenario	2019	Patients with stage I breast, colorectal and lung cancer and T1 melanoma	Australia	Impact of cancer care delays
Dolan *et al.* [18]	Retrospective, single centre	Group 1: baseline (2019)—375 patients Group 2: pandemic (March–May 2020)—58 patients	2019–2020	Patients with NSCLC lung cancer	USA	Thoracic oncologic surgical activity
Englum *et al.* [9]	Retrospective, multicentre	Group 1: baseline (2018–2019) Group 2: pandemic (2020)	2018–2020	Patients with prostate, lung, bladder and colorectal cancer	USA	Cancer-related health care encounters, diagnostic or screening procedures, new cancer diagnoses
Flores *et al.* [8]	Retrospective, single centre	Monthly case average compared between baseline (2018–2019) to the pandemic period (2020–2021)	2018–2021	Patients with lung cancer	USA	Rates of lung cancer diagnosis, disease stage
Fu *et al.* [32]	Retrospective, single centre	Group 1: baseline (2015–2019)—2054 patients Group 2: pandemic (2020)—397 patients	2015–2020	Patients with lung cancer	China	Lung cancer diagnosis; subjective patient outcomes
Fujita *et al.* [33]	Retrospective, single centre	Group 1: no treatment delay—150 patients Group 2: treatment delay—15 patients	2020	Patients with lung cancer	Japan	Lung cancer treatment; subjective patient outcomes
Geukens *et al.* [45]	Retrospective, multicentre	928 patients	2020–2021	Patients with malignancy	Belgium	Mortality
Gokce *et al.* [41]	Retrospective, single centre	35 patients	2020–2021	Patients with NSCLC lung cancer	Turkey	Lung cancer surgery outcomes
Hilzenrat *et al.* [15]	Observational, multicentre	Electronic survey distributed to CATS members	2019–2020	Patients with lung cancer	Canada	Surgical lung cancer care
Kasymjanova *et al.* [20]	Retrospective, single centre	Group 1: baseline (March 2019–February 2020) Group 2: pandemic (March 2020–February 2021)	2019–2021	Patients with lung cancer	Canada	Lung cancer diagnosis and treatment
Keogh *et al.* [21]	Retrospective, single centre	Group 1: baseline (January 2019–February 2020)—330 patients Group 2: pandemic (March 2020–February 2021)—320 patients	2019–2021	Patients with NSCLC lung cancer	Canada	Lung cancer diagnosis, stage, treatment, outcomes
Leclère *et al.* [40]	Retrospective, single centre	115 patients	2020	Patients with NSCLC lung cancer undergoing surgical resection	France	Postoperative occurrence of COVID-19
London *et al.* [17]	Retrospective, multicentre	Group 1: baseline (January–April 2019) Group 2: pandemic (January–April 2020)	2019–2020	Patients with malignancy; CCRN database	USA	Rates of lung cancer diagnosis
Lou *et al.* [12]	Retrospective, single centre	Group 1: pre-pandemic period (2016–2019) Group 2: pandemic (2020)	2016–2020	Patients undergoing screening for lung, colorectal and breast cancer	USA	Cancer-related screening and diagnosis
Malagón *et al.* [48]	Population-based modeling study	Model of cancer incidence, stage at diagnosis and survival	2020–2021	Patients with malignancy; Canadian Cancer Registry	Canada	Impact of cancer care delays
Maringe *et al.* [49]	Population-based modeling study	29 305 patients	2010–2015	Patients with breast, colorectal, esophageal and lung cancer; NHS database	UK	Impact of cancer care delays
Martínez-Hernández *et al.* [37]	Retrospective, multicentre, questionnaire	Electronic survey sent to SPECT members	2019–2020	Patients with lung cancer	Spain	Lung cancer management
Morais *et al.* [26]	Retrospective, multicentre, propensity-matched	Group 1: baseline (March–July 2019) Group 2: pandemic (March–July 2020)	2019–2020	Patients with malignancy	Portugal	Lung cancer management
Morais *et al.* [27]	Retrospective, single centre	Group 1: baseline (March–July 2019) Group 2: pandemic (March–July 2020)	2019–2020	Patients with malignancy	Portugal	Cancer-related screening and diagnosis
Pasello *et al.* [30]	Retrospective, multicentre	Group 1: baseline (March–April 2019) Group 2: pandemic (March–April 2020)	2019–2020	Patients with lung cancer; two centres	Italy	Lung cancer management
Patt *et al.* [7]	Retrospective, multicentre	Group 1: baseline (March–July 2019) Group 2: pandemic (March–July 2020)	2019–2020	Patients with malignancy; Medicare FFS claims	USA	Cancer-related screening and diagnosis
Peacock *et al.* [24]	Retrospective, multicentre	Group 1: baseline (April 2019) Group 2: pandemic (April 2020)	2019–2020	Patients with malignancy; Belgian Cancer Registry	Belgium	Rates of lung cancer diagnosis
Peer *et al.* [42]	Retrospective, single centre	113 patients	2020	Patients with NSCLC lung cancer undergoing surgical resection	Israel	Lung cancer surgical outcomes
Piwkowski *et al.* [25]	Retrospective, multicentre	Group 1: baseline (2019) Group 2: pandemic (2020)	2019–2020	Patients with lung cancer undergoing surgery; PNLCR database	Poland	Lung cancer stage and surgical outcomes
Priou *et al.* [28]	Retrospective, multicentre	Group 1: baseline (2018–2019) Group 2: pandemic (2020)	2018–2020	Patients with lung cancer	France	Lung cancer diagnosis, stage, treatment and outcomes
Ramanakumar *et al.* [22]	Retrospective, multicentre	Group 1: baseline (averages of 2018, 2019, 2020) Group 2: pandemic (April 2020–March 2021)	2018–2021	Patients with lung, breast, colorectal and prostate cancer	Canada	Rates of newly diagnosed malignancy
Sato *et al.* [16]	Retrospective, multicentre	Group 1: baseline (2014–2019) Group 2: pandemic (2020)	2014–2020	Patients with thoracic malignancy	Japan	Lung cancer diagnosis, stage, treatment and outcomes
Seitlinger *et al.* [41]	Prospective, multicentre	731 patients	2020	Patients undergoing thoracic oncologic surgery	France, Germany, Italy, Canada	Thoracic oncologic surgery outcomes
Sha *et al.* [3]	Retrospective, single centre	Total patients: 161	2020	Patients with lung cancer	China	Delays in cancer-related care
Shipe *et al.* [50]	Population-based modeling study	Immediate versus delayed surgical resection	–	Patients with suspicious lung nodules <2 cm	USA	Impact of cancer care delays; 5-year survival
Sud* et al.* [51]	Population-based modeling study	Model of cancer progression during COVID-19-induced delays in care	2013–2017	Patients with malignancy	UK	Hazard ratios of cancer progression, 5-year reduction in survival
Taylor *et al.* [36]	Observational, interview and questionnaire based	30 patients	2020	Patients with lung cancer	UK	Lung cancer quality of care and quality of life
Terashima *et al.* [34]	Retrospective, single centre	Group 1: baseline (March 2018–March 2019)—82 patients Group 2: pandemic (March 2020–March 2021)—75 patients	2018–2021	Patients with lung cancer	Japan	Lung cancer pre-visit, pre-diagnosis and pre-treatment time
Teteh *et al.* [35]	Observational, single centre	41 participants	2020	Patients enrolled in a randomized trial of self‐management intervention for lung cancer surgery preparation/recovery	USA	Subjective outcomes of lung cancer surgery preparation/recovery
Uematsu *et al.* [43]	Observational, questionnaire-based	Questionnaire sent to 14 facilities in the Kanagawa General Thoracic Surgery Study Group	2020	Patients with lung cancer	Japan	Lung cancer diagnosis and resource allocation
Van Haren *et al.* [12]	Retrospective, single centre	Group 1: baseline (January 2017–February 2020) Group 2: pandemic (March–July 2020)	2017–2020	Patients undergoing screening for lung cancer	USA	Cancer-related screening and diagnosis
Villena-Vargas *et al.* [19]	Retrospective, single centre	Group 1: baseline (January 2020–March 2020)—57 patients Group 2: pandemic (March 2020–June 2020)—42 patients	2020	Patients with NSCLC lung cancer	USA	Outcomes of lung cancer surgery
Walker *et al.* [14]	Retrospective, multicentre	Group 1: baseline (2019) Group 2: pandemic (2020)	2019–2020	Patients undergoing screening for breast, cervical, colorectal and lung cancer	Canada	Cancer-related screening and diagnosis
Walter *et al.* [31]	Observational, questionnaire-based	Questionnaire sent to 245 patients	2020	Patients with thoracic malignancy	Germany	Subjective delays in thoracic malignancy diagnosis and treatment
Yu *et al.* [46]	Retrospective, single centre, propensity-matched	Group 1: baseline (September 2019–November 2019) Group 2: pandemic (December 2019–February 2020)	2019–2020	Patients with NSCLC lung cancer	China	Lung cancer treatment and outcomes
Zhang *et al.* [6]	Retrospective, single centre	Group 1: baseline (February–July 2019) Group 2: pandemic (February–July 2020)	2019–2020	Patients with lung cancer	China	Cancer-related screening and diagnosis

CATS: Canadian Association of Thoracic Surgeons; CCRN: COVID and Cancer Research Network; COVID: coronavirus disease 2019; FFS: fee for service; NHS: National Health Service; NSCLC: non-small-cell lung cancer; PNLCR: Polish National Lung Cancer Registry; RCRD: Rapid Cancer Registration Dataset; SPECT: Spanish Thoracic Surgery Society.

## RESULTS

### Impact of COVID-19 on lung cancer screening

In an analysis of the Veteran Affairs Healthcare System in the USA, Englum *et al.* reported an overall decrease in new cancer diagnoses by 13% and a 10% decrease in screening chest computed tomography (CT) scans [9]. An analysis of the Medicare fee-for-service population from 2019–2020 indicated a 74% and 58% reduction in lung cancer screening and lung biopsy rates at the peak of the pandemic, respectively [7]. Furthermore, Van Haren *et al.* noted a significant increase in the ‘no-show rate’ and a decrease in the number of low-dose CT scans for patients scheduled for lung cancer screening. Subsequently, there was a significant increase in lung nodules suspicious for malignancy (Lung-RADS 4) upon resumption of screening [12]. Interestingly, 1 retrospective, single-centre study from the USA reported no difference in screening chest CTs during the pandemic period [13].

A multicentre study in Canada reported a 23% absolute reduction in screening and an increased rate of higher Lung-RADS categories in January–March 2020 compared to baseline (16.7% vs 12.8%) [14]. Additionally, a survey completed by 59% of the Canadian Association of Thoracic Surgeons reported essentially unchanged access to CT during the pandemic. However, 87%, 85% and 63% of surgeons reported ‘somewhat less’ or ‘much less’ access to flexible bronchoscopy, linear endobronchial ultrasound and pulmonary function tests, respectively [15].

Finally, a retrospective, multicentre study in Japan analysing patients with thoracic malignancies also reported a reduction in lung cancer screening [16].

### Impact of COVID-19 on lung cancer rates & presentation

An analysis of the COVID and Cancer Research Network in the USA between 2019 and 2020 indicated a 39.1% reduction in new lung cancer diagnoses [17]. Flores *et al.* reported a 50% reduction in the proportion of stage 0/I/II lung cancers and a 179% increase in the proportion of stage III/IV lung cancers during the first lockdown. Similarly, they note a further 50% decrease and a 130% increase for the same during 2021 [8]. Additionally, a retrospective, single-centre US study indicated significantly increased tumour size at presentation during the pandemic [18]. Lou *et al.* [13] reported a significant decrease in new lung cancer diagnoses in 2020 but, interestingly, there was no change in stage at presentation. Similarly, in their retrospective, single-centre analysis of patients with non-small-cell lung cancer (NSCLC), Villena-Vargas *et al.* [19] also reported no significant difference in tumour pathologic stage at presentation.

One Canadian study indicated a 35% decrease in new lung cancer diagnoses along with an increased stage at diagnosis during the pandemic [20]. An analysis of solely NSCLC patients at a different Canadian centre indicated no significant difference in the number of patient referrals, baseline patient characteristics or tumour pathologic state [21]. Another Canadian centre reported a 5–34% underestimation of the incidence of stage-specific lung cancer during the first wave of the pandemic corresponding to the temporary suspension of local screening programs [22].

In Italy, Cantini *et al.* [5] reported a reduction in the number of newly diagnosed cases of lung cancer during the pandemic as well as an increased likelihood of stage IV disease. Another Italian study indicated a significant increase in the number of patients with IB–IIB disease and no difference in the number of patients with advanced-stage disease [23].

An analysis of the Belgian Cancer Registry from 2019 to 2020 indicated a 33% reduction in new lung cancer diagnoses [24]. The Polish National Lung Cancer Registry noted an increased rate of patients with more advanced pathological stage during the pandemic [25]. Similarly, a retrospective analysis of a single centre in Portugal reported a 21.5% reduction in new lung cancer diagnoses and an increased diagnosis of advanced-stage cancer [26]. A propensity-matched multicentre study in Portugal also indicated a 20% reduction in new lung cancer diagnoses [27].

The results of the National Lung Cancer Audit (NLCA) in the UK indicated a 4% absolute increase in patients with lung cancer presenting emergently. However, contrasting with some of the previous results, there was no difference in the proportion of patients with advanced (stage IV) disease [2]. A retrospective, multicentre analysis in France also indicated a 32% decrease in new lung cancer diagnoses but no significant difference in the initial tumour stage [28].

In China, Zhang *et al.* [6] reported that 47.6% of lung cancers were diagnosed incidentally, 34.2% based on symptoms and 18.2% physical exam during the pandemic, with the average tumour volume of the patients diagnosed by screening significantly smaller than in those diagnosed by symptoms or physical exam. A retrospective, multicentre study in Japan analysing patients with thoracic malignancies reported a 3.2% increase in the number of metastatic lung tumour surgeries during the pandemic [16].

### Delays in lung cancer care during the COVID-19 pandemic

A multicentre study from the USA reported that delays in imaging and diagnostic procedures were more likely with lung cancer than other solid tumours and hematological malignancies [29]. Interestingly, in another US study, Lou *et al.* [13] reported a reduced time-to-treatment for lung cancer during the pandemic, with more patients undergoing surgical resection.

The analysis of patients with lung cancer at a single centre in Canada did not report delays in the initiation of chemotherapy and radiation treatment; however, they did note increased delays to surgical intervention [20].

In Italy, Pasello *et al.* reported reductions in outpatient visits, patient enrolment in clinical trials and end-of-life cancer systemic treatments, as well as increased tumour processing times during the pandemic [30]. However, another Italian study reported no differences in the interval between symptom onset and radiological/cytohistological diagnosis, treatment start and first radiological revaluation [5]. Similarly, the NLCA in the UK reported no difference in median time from diagnosis to treatment between 2019 and 2020 [2].

In a survey sent to 245 patients in Germany, 57–70% reported unavailability of respiratory/physical therapy, as well as delays or cancellations of appointments for tumour‐directed therapy, imaging and follow‐up care in 18.9%, 13.6% and 14.8% of patients, respectively [31].

In a single-centre analysis of 161 patients in China, delayed follow-up care and discontinued or delayed cancer treatment were noted in 59% and 9% of patients, respectively. Eighteen percent of patients with delayed care exhibited progressive disease upon reassessment [3]. In a nationwide survey, Fu *et al.* [32] reported that patient concerns included long waiting times for outpatient services, inpatient beds, physical examinations or operations.

The results of a single-centre analysis in Japan noted pandemic-related delays in lung cancer treatment in 9.1% of patients [33]. Similarly, Terashima *et al.* [34] identified increases in pre-visit time, surgery wait time, as well as reductions in surgery for lung cancer and increased rates of advanced disease.

Teteh *et al.* [35] surveyed patients and caregivers on their perspectives on care during the pandemic, identifying themes of delayed treatment, isolation, psychological distress and financial burden. In addition to that noted above, another survey reported mixed feelings regarding virtual care [36].

### Surgical intervention for lung cancer during the coronavirus disease 2019 pandemic

A retrospective, single-centre analysis of lung cancer surgical outcomes in the USA reported no significant differences in postoperative complications, 30, 60 or 90-day mortality, between pre-pandemic and pandemic patient populations [18]. In their analysis of American patients with NSCLC, Villena-Vargas *et al.* reported no significant difference in 90-day postoperative morbidity or mortality. However, there was a 40% mortality rate associated with COVID-19 infections within 90 days of surgery [19].

A Canadian study reported increased utilization of radiosurgery as the first definitive treatment with a corresponding decrease in systemic treatment and surgery [20]. This was as a result of recommendations to utilize available treatments as alternatives to surgery. The reductions in surgical capacity were attributed to reductions in operating room hours, lack of medical staff and long waiting lists for surgery. An analysis of solely NSCLC patients at another Canadian site reported no difference in surgical mortality or hospital length of stay during the pandemic as compared to the pre-pandemic baseline [21].

The NLCA in the UK reported a 5% absolute reduction in patients with NSCLC undergoing surgical intervention [2]. Survey results of the Spanish Society of Thoracic Surgeons indicated a 96% reduction in thoracic surgical activity between 2019 and 2020 [37]. In their description of thoracic oncological surgical activity in multiple centres in Italy, Ciriaco *et al.* note a 50% reduction in thoracic surgery beds during the pandemic but no decrease in the number of surgical procedures. They describe a ‘hub and spokes’ approach to surgical care for patients with NSCLC [38]. The hub and spoke model refers to the establishment of ‘spoke’ centres where patients are initially seen and evaluated, following which they can be referred to a central ‘hub’ which is specialized in the treatment of lung cancers and ideally COVID-free to prevent interruptions of care. A similar hub and spoke model was described by Bertolaccini and Spaggiari [39] in the management of lung cancer in the Lombardy region of Italy. In a retrospective analysis of 115 patients with NSCLC undergoing surgical resection at a single centre in France, Leclère *et al.* report no immediate difference in oncologic outcomes. The rate of postoperative COVID-19 infection at 1 month was 5%. In patients with postoperative COVID-19 infection, they did observe an increased rate of readmission, but no significant increase in 30-day morbidity or mortality [40]. An analogous study consisting of 35 NSCLC patients undergoing surgical resection at a single centre in Turkey from 2020 to 2021 reported 0% and 14% rates of postoperative mortality and morbidity, respectively. There was no significant difference in complications in patients with a preoperative history of COVID-19 [41]. Finally, utilizing the Polish National Lung Cancer Registry, Piwkowski *et al.* [25] reported a 20.5% overall reduction in the number of lung cancer procedures, with no difference in rates of in-hospital mortality and decreased hospital length of stay during the pandemic.

A retrospective analysis of 113 patients with NSCLC undergoing surgical resection at a single centre in Israel noted postoperative complications slightly higher than other studies at 30.9% [42].

In a survey sent to 14 facilities in the Kanagawa General Thoracic Surgery Study Group, Uematsu *et al.* [43] reported a 22% reduction in total surgical case volume with a 6% and 14% reduction in lung cancer surgery at all institutions and those placed under restriction, respectively. A similar study carried out in China reported a 57% reduction in lung cancer surgery during the pandemic.

Finally, a multicentre study consisting of 731 patients undergoing thoracic oncologic surgery in France, Germany, Italy and Canada, reported 3%, 1.2% and 0.5% rates of overall mortality, COVID-19 infection and readmission [44].

### Lung cancer outcomes during the coronavirus disease 2019 pandemic

According to the NLCA in the UK, there was a reduction in median survival from 2019 to 2020 (306 vs 316 days) [2].

In a retrospective, study consisting of 928 patients at 19 different hospitals across Belgium, there was a trend towards higher mortality in patients with lung cancer as compared to other solid organ cancers (27.6% vs 20.8%, *P* = 0.062) [45].

However, a retrospective, propensity-matched study in Portugal from 2019 to 2020 indicated no difference in crude, age-adjusted, stage-adjusted and propensity-matched hazard ratios for short-term mortality with lung cancer treatment overall during the pandemic. Of note, a significantly higher hazard ratio for mortality was observed in patients with stage III cancer [27].

A retrospective, multicentre analysis of patients with lung cancer in France over the same period also reported no significant difference in rates of 6-month mortality following systemic anticancer therapy or surgical intervention. However, they did note increased mortality in patients with COVID-19 who received systemic anticancer therapy [28].

In a propensity-matched study of 196 patients with NSCLC in China between 2019 and 2020, Yu *et al.* noted an increased rate of therapy discontinuation and non-surgical treatment for late-stage patients, but no difference in surgical treatment, discontinued therapy, cancer progression and mortality for early-stage patients [46].

### Modelling of the impact of COVID-19-related delays in lung cancer care

In a population-based modelling study by Degeling *et al.*, there is an estimated 8.3% and 16.0% probability of cancer progression with 3- and 6-month treatment delays, respectively. Three- and 6-month delays in care are estimated to result in 11 and 43 excess deaths, as well as 98 and 373 life-years lost at 10 years, respectively [47].

A similar study focusing on the Canadian population estimated a 1.1% relative mortality increase with 3082 excess deaths and 42 472 life-years lost due to the pandemic-related diagnostic and treatment delays for lung cancer [48].

An analysis from the UK estimated a 4.8–5.3% increase in lung cancer mortality 5 years after diagnosis [49].

Interestingly, an American study analysing the impact of immediate versus delayed (>3 months from diagnosis) surgical resection in patients with suspicious lung nodules <2 cm estimated no significant difference in 5-year survival for the overall patient population. However, survival is estimated to be significantly greater with delayed resection if the probability of COVID-19 infection is >13% [50].

### Limitations

The literature published to date on resource allocation and outcomes during the COVID-19 pandemic is variable with heterogeneous studies and trends identified. Additionally, while negative implications for postoperative outcomes were not consistently identified, this may have been due to insufficient follow-up to date requiring longer-term studies on the outcomes of patients with lung cancer as the pandemic progresses. Furthermore, relatively few studies published data on the outcomes of these patients making it difficult to identify trends in the outcomes of these patients. Follow-up and presentation of the data for patients with lung cancer identified during or after the pandemic will be required to identify the impact on their outcomes over the long term.

## CONCLUSIONS

This systematic review aimed to summarize the literature investigating the impact of resource allocation towards the COVID-19 pandemic on patients with lung cancer. Several interesting trends were identified in the literature published to date. First, rates of screening and diagnostic testing were reduced during the pandemic secondary to reduced access to these resources [7, 9, 12–16]. Second, investigations across the literature identified mixed results in the presentation of patients with lung cancer with several studies identifying decreased incidence of early-stage lung cancer, with a correlated increased incidence of late-stage disease [2, 5, 8, 13, 17–28]. Patients found to have lung cancer during the pandemic experienced reduced rates of in-person assessments, increased time to intervention and reduced follow-up [3, 13, 20, 29–33]. These differences in findings are likely multifactorial including the fact that many of these studies were published in different regions of the world where the impact and timing of the various waves of the pandemic differed, there were differing responses to the pandemic from governments and healthcare authorities, and there are diverse healthcare systems with contrasting priorities and resource utilization. Among patients undergoing surgical intervention for lung cancers during the pandemic, there were few differences identified in postoperative outcomes [18, 19, 21, 25, 28, 40–42, 45, 46]. Finally, when accounting for the above disruptions in screening, assessments, diagnostics and interventions, studies aimed at modelling the impact of these changes predict significant increases in mortality for patients with lung cancer in the years to come [47–50].

The findings of this review highlight the consequences of changes in healthcare resource allocation during the COVID-19 pandemic. The studies identified in this review highlight the impact of the pandemic on screening, diagnostic testing and surgical interventions, but interestingly, did not identify consistently significantly worse outcomes for patients [7–9, 13–16, 18, 20, 22]. While the pandemic had implications over the past 2 years on screening and diagnosis, the outcomes included in many of the published studies did not differ significantly. Reduced screening in the first year of the pandemic may have affected the incidence of lung cancer but not postoperative outcomes as many cases of lung cancer would have been undiagnosed. However, the indirectly related delayed presentation will affect morbidity and mortality in the years to come. As previously asymptomatic cases progress we will likely see a shift in staging to more advanced lung cancer. This stage shift will not only affect lung cancer survival rates but shift care from surgery for early-stage cancer to either multimodality treatment protocols or palliation further magnifying the impact of the COVID-19 pandemic on lung cancer resources in the future years. Due to these factors, a relatively small number of studies published data on outcomes of patients. This is likely related to the prolonged course of lung cancer developing over months or years. While we are able to appreciate differences in screening and management, data on outcomes will require years to clearly identify. Future investigations focusing on the outcomes of these patients will be required to follow their progress over time.

Given the recently identified impacts on patient outcomes and the significant implications for patients with lung cancer in the future, several considerations are required in the late stages of this pandemic and in future scenarios where resources may be scarce. While the pandemic certainly required mobilization and diversion of significant healthcare resources, an over-emphasis on the pandemic without concurrent consideration of non-COVID patients, such as those with lung cancer, may result in lasting impacts on those patients. As patients with lung cancer develop more advanced disease, prolonged treatment with worse prognoses will likely result in additional strain on healthcare resources. Referral to evidence based and guideline recommendations may help to identify key areas that are pivotal to the management of patients with lung cancer, and other conditions. While care during the pandemic in the included studies was infrequently compared to guideline recommendations for screening or management, the delay or cessation of screening and management of lung cancer likely delayed care well past guideline recommendations in many cases. As we are able to retrospectively analyse the efficacy of resource allocation and the unintended side effects, in future times when resources are scarce the implications of resource diversion must be considered for all patients to ensure optimal use of resources. This will mitigate the long-term effects of resource diversion and reallocation preventing further draining of healthcare resources to treat potentially preventable suboptimal outcomes.

Through reflecting on the pandemic response, we are able to understand the implications of specific decisions in order to guide future decision-making in times of limited resources. Future decisions regarding resource allocations should consider the impact on all patients, including the long-term results of progressive diseases not being identified or treated optimally. These decisions should be guided by past trends to provide optimal care to all patients.

As the COVID-19 pandemic placed enormous strains on global healthcare systems, resource allocation away from most sectors of healthcare to battle the pandemic was required. While significant resources were necessary to combat the pandemic, the shift of resources away from other areas of healthcare has had detrimental impacts on non-COVID patients. The findings of this review suggest that patients with lung cancer have been shown to experience reduced rates of screening, diagnosis and interventions during the pandemic. While significant differences in postoperative outcomes were not identified, the effects of the pandemic and reductions in cancer screening will likely be identified in the years to come. Future consideration of the long-term implications of resource allocation away from patients with lung cancer with an attempt to provide equitable access to healthcare and to limit the interruptions of patient care may help provide the best care for all patients during times of limited resources.

## Data Availability

The data that supports the findings of this study are available within the article and its supplements.

## ABBREVIATIONS

COVID-19: Coronavirus disease 2019
CT: Computed tomography
NLCA: National Lung Cancer Audit
NSCLC: Non-small-cell lung cancer
